# Carrot Rhamnogalacturonan-I Supplementation Shapes Gut Microbiota and Immune Responses: A Randomised Trial in Healthy Adults

**DOI:** 10.3390/microorganisms13092156

**Published:** 2025-09-16

**Authors:** Evangelia N. Kerezoudi, Sue McKay, Seta Kurt, Maaike De Kreek, Jelle De Medts, Lynn Verstrepen, Jonas Ghyselinck, Lieven Van Meulebroek, Wim Calame, Annick Mercenier, Ruud Albers, Robert J. Brummer, Ignacio Rangel

**Affiliations:** 1 Nutrition-Gut-Brain Interactions Research Centre, School of Medical Sciences, Örebro University, 70182 Örebro, Sweden; robert.brummer@oru.se (R.J.B.); ignacio.rangel@oru.se (I.R.); 2 NutriLeads BV, Bronland 12-N, 6708 WH Wageningen, The Netherlands; sue.mckay@nutrileads.com (S.M.); annick.mercenier@nutrileads.com (A.M.); ruud.albers@nutrileads.com (R.A.); 3 Department of Clinical Research Laboratory, School of Medical Sciences, Faculty of Medicine and Health, Örebro University, 70182 Örebro, Sweden; seta.kurt@regionorebrolan.se; 4 Clinical and Experimental Endocrinology, KU Leuven, Herestraat 49 ON1bis, Box 902, 3000 Leuven, Belgium; maaike.dekreek@kuleuven.be; 5 ProDigest BV, Technologiepark 82, 9052 Zwijnaarde, Belgium; jelle.demedts@prodigest.eu (J.D.M.); jonas.ghyselinck@prodigest.eu (J.G.); 6 Laboratory of Integrative Metabolomics, Department of Translational Physiology, Infectiology and Public Health, Faculty of Veterinary Medicine, Ghent University, Salisburylaan 133, 9820 Merelbeke, Belgium; lieven.vanmeulebroek@prodigest.eu; 7 StatistiCal BV, Strandwal 148, 2241 MN Wassenaar, The Netherlands; w.calame@kpnplanet.nl

**Keywords:** prebiotic, healthy adults, rhamnogalacturonan-I, cRG-I, bifidobacteria, DC activation, diarrhoea score

## Abstract

**Background:** Rhamnogalacturonan-I (RG-I) is a pectic polysaccharide with emerging prebiotic and immunomodulatory potential. This randomised, double-blind, placebo-controlled trial (ID: NCT06081972) evaluated the effects of carrot-derived RG-I (cRG-I) supplementation, compared to placebo (maltodextrin), on gut microbiota composition and immune cell activation in healthy adults. **Methods:** A total of 54 participants (18–70 years old) were randomised in a double-blind manner to receive either 500 mg/day of cRG-I or placebo for four weeks. Pre-screening ensured balanced randomisation based on habitual fibre intake and faecal *Bifidobacterium* counts. Questionnaires assessed potential gut health and well-being effects, while in vitro and ex vivo models were used to evaluate effects on intestinal permeability. **Results:** cRG-I was well tolerated with excellent compliance. Faecal *Bifidobacterium* counts increased significantly, peaking at week 3. Isobutyric acid levels rose, though no other SCFAs differed. Immunologically, cRG-I enhanced the percentage of circulating myeloid dendritic cells expressing activation markers (CD86, HLA-DR) on. Stool consistency improved slightly. Preclinical models further showed that cRG-I and its fermentation products protected intestinal barrier integrity under stress. **Conclusion:** These results support cRG-I as a safe, low-dose dietary intervention capable of beneficially modulating gut microbiota, immune responses, and barrier function in healthy adults within a short supplementation period.

## 1. Introduction

Dietary fibres play a crucial role in disease prevention, with a high fibre diet being associated with a reduced risk of developing cardiovascular diseases, diabetes, obesity, colorectal cancer [1,2]. However, declining fibre consumption, particularly in populations adhering to a Western-type diet, has become a growing public health concern. Dietary fibre intake generally falls below the recommended 25 g/day for adults [3]. Insufficient fibre intake depletes fermentable substrates available to the gut microbiota [4], which can lead to the loss of specific bacterial species, a decline in microbial diversity, and a reduction in beneficial fermentation-derived metabolites, such as short-chain fatty acids (SCFAs) [5]. While typical dietary recommendations focus on increasing overall fibre intake, recent research shows that not all fibres have the same effect, and some fibres, such as carrot-derived rhamnogalacturonan-I (cRG-I), even provide benefits at lower doses [6,7].

Some dietary fibres have emerged as modulators of gut microbiota composition and function [8]. Soluble fibres undergo fermentation by gut bacteria, primarily in the colon, leading to the production of bioactive microbial metabolites such as SCFAs, including acetate, propionate, and butyrate. Acetate and propionate serve as energy substrates for other gut bacteria and can also act systemically, supplying energy to various tissues and organs, including the gut epithelium, muscle, and brain. Butyrate, in particular, is a crucial energy source for colonocytes and is well-documented for its anti-inflammatory and immunomodulatory properties [9,10]. Beyond metabolite production, dietary fibres also influence gut microbiota composition. A diverse gut microbiota is associated with better health outcomes, including enhanced intestinal barrier integrity and improved resilience against pathogens [11,12]. Additionally, some dietary fibres are increasingly recognised as regulators of the immune system, exerting effects through both direct and indirect mechanisms [13]. Direct interactions occur between fibres and immune cells, such as dendritic cells (DCs) in Peyer’s patches of the small intestine, through pattern recognition receptors (PRRs). Indirectly, microbial metabolites such as SCFAs mediate immune modulation by influencing immune cell responsiveness [14]. Among the diverse bacterial taxa in the gut, members of the genus *Bifidobacterium* are of particular relevance for host health. They are among the earliest colonisers of the human intestine and remain important constituents of the adult microbiota, where they contribute to carbohydrate fermentation, SCFA production, and cross-feeding interactions with other microbes [15]. Beyond their metabolic roles, bifidobacteria are frequently associated with beneficial effects on gut barrier function [16,17], immune regulation [18,19], and protection against pathogens [20,21].

With the knowledge that the diet is potentially responsible for (up to) 20% of the variation in adult gut microbiota composition [22,23], dietary interventions represent an interesting strategy to modulate the gut microbiota composition and the production of active metabolites like SCFAs, which can subsequently influence health outcomes. For these reasons, an increase in bifidobacteria is commonly considered a hallmark of prebiotic activity and a meaningful endpoint in dietary intervention studies. Intervention studies with dietary fibres, particularly prebiotics, have demonstrated benefits across various health domains, including improved mineral absorption, enhanced gut barrier function, weight management, metabolic health and mental health [22,24,25,26]. Recent studies have highlighted the immunomodulatory potential of a pectic polysaccharide derived from carrot (cRG-I). Supplementation with 300 mg/day of cRG-I for eight weeks in healthy adults significantly reduced the severity (by ~20–33%) and duration (by ~25–43%) of common cold symptoms, and accelerated innate antiviral and interferon responses, including faster induction of interferon-stimulated genes and enhanced NK cell cytotoxic activity. These responses were associated with faster viral clearance and improved quality of life [27,28]. These effects were also accompanied by an increased relative abundance of *Bifidobacterium* spp., particularly *B. longum* and *B. adolescentis* [7]. Preclinical studies using in vitro and ex vivo faecal fermentations with cRG-I have further demonstrated modulation of the gut microbiota including consistent bifidogenic effects [29,30,31], increased SCFA production [32], and gut barrier protective properties [29].

Building on this existing evidence, the present study aimed to investigate whether a shorter supplementation period (4 weeks) with a slightly higher dose (500 mg/day) of cRG-I—within the effective range previously shown to induce microbiota and immune benefits (300 and 1500 mg/day) [7,27,28]—could elicit microbiota-modulating and immunomodulatory effects in healthy individuals. The primary objective was to assess whether a bifidogenic response could be achieved within four weeks by tracking changes in *Bifidobacterium* spp. counts every week. Secondary outcomes included measurements of faecal SCFAs, faecal calprotectin levels, and activation markers on circulating immune cells. Additionally, validated questionnaires were used to evaluate potential effects on gut health and well-being. To further explore the mechanistic basis of these effects, preclinical in vitro and ex vivo models were employed to assess gut barrier integrity and immune cell activation. We hypothesised that cRG-I supplementation would promote the growth of beneficial bacteria, particularly *Bifidobacterium* spp., leading to increased production of bioactive metabolites. While the in vivo intervention focused on quantifying bifidobacteria and related outcomes, complementary preclinical models were used to directly assess whether fermented cRG-I itself could protect intestinal barrier integrity under stress conditions.

## 2. Materials and Methods

### 2.1. Human Dietary Intervention

#### 2.1.1. Study Design and Allocation Procedure

**Figure 1:**
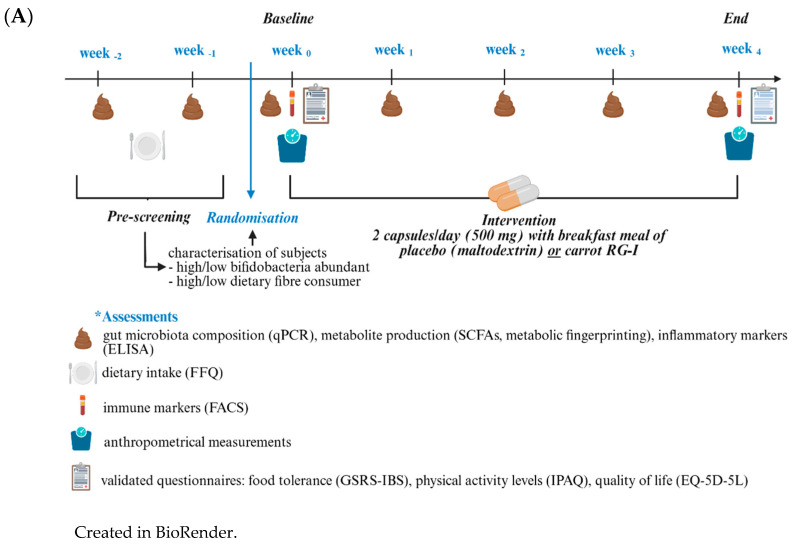

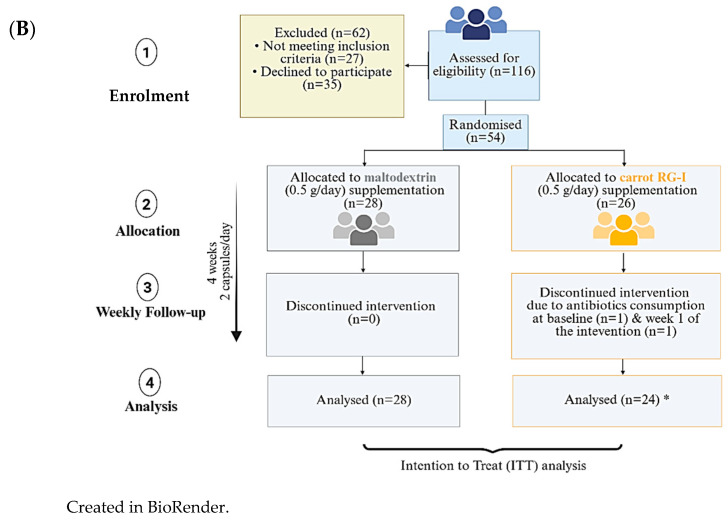
(**A**) Study design and (**B**) subjects’ disposition. * The number of individuals included in the analysis corresponds to those actively participating in the study at each specific timepoint. At baseline, the total number of participants was n = 26. Created in BioRender. https://BioRender.com/b97z315 (accessed on 30 July 2025); https://BioRender.com/m49u742 (accessed on 30 July 2025).

This randomised, double-blind, placebo-controlled parallel design study was conducted between September 2023 and March 2024 at Örebro University, Sweden and was organised into three cohorts based on initial enrolment timing (Figure 1A). Data for average daily dietary fibre intake and baseline faecal *Bifidobacterium* spp. levels was collected during a two-week pre-intervention phase and participants meeting the eligibility criteria were stratified as either high or low fibre consumers (≥ or <22.66 g/day fibre intake) and as high or low bifidobacteria carriers (≥ or <0.894 log *Bifidobacterium* copies/µL, normalised to a DNA concentration of 100 ng/µL), based on the median values obtained from the first cohort of participants. This stratification was carried out by a researcher blinded to group allocation, primarily to optimise randomisation efficiency and ensure balanced distribution of key host and microbial factors across the two supplementation groups. Given the sample size, stratification was not intended to support powered subgroup analyses but served as a basis for exploratory post hoc assessments. Further on, a computational randomisation algorithm allocated participants to either the carrot-derived RG-I (cRG-I; 500 mg per day) or the placebo (maltodextrin) group, and participants were instructed to take the test article (two capsules/day) with breakfast over a four-week period. Participants were encouraged to maintain their typical diet and lifestyle throughout the supplementation period. At the end of the study, participants returned the remaining test article to assess compliance. The group assignments were concealed from both investigators and participants throughout the study, with unblinding occurring exclusively following the completion of data analysis.

The study adhered rigorously to Good Clinical Practice standards and conformed entirely to the ethical stipulations of the Declaration of Helsinki. Informed consent documents describing the study’s aims and procedures were provided and signed by all participants. The Swedish Ethical Review Authority in Uppsala approved the study (Dnr. 2023-03938-01) and it was registered at ClinicalTrials.gov (ID: NCT06081972) https://clinicaltrials.gov/study/NCT06081972?term=NCT06081972&rank=1 on 20 September 2023.

Participants were eligible if they were between 18–70 years old, had a Body Mass Index (BMI) between 18 and 30 kg*m^−2^ and were willing to refrain from using supplements (i.e., probiotics, prebiotics, symbiotics) that could affect gastrointestinal function for four weeks prior to, and during, study visits (Figure 1B). Exclusion criteria included a history of complex gastrointestinal surgeries or disorders, psychiatric diseases and the use of systemic antibiotics, corticosteroids, laxatives, anti-diarrhoeal drugs, or anticholinergics within three months before the study. Additionally, regular use of non-steroidal anti-inflammatory drugs (NSAIDs) in the two months prior to the study, smoking or snuff use within three months, and substance abuse (alcohol and drugs) were grounds for exclusion. Individuals following vegan diets, pregnant or breastfeeding women, and those with a recent weight change exceeding 5% of their average body weight in the month prior to the study were also excluded.

#### 2.1.2. Test Articles

cRG-I is a natural extract from carrot (*Daucus carota* subsp. *sativus*). The patented ingredient, known as Benicaros^®^, supplied by NutriLeads (Wageningen, The Netherlands) is a water-soluble nondigestible fermentable fibre enriched (80%) in the RG-I domain of pectin. The extraction method and extract characteristics (composition and structure) have been described previously [33]. Test articles were prepared as hydroxypropylmethylcellulose (HMPC) capsules by Lab-medisan (Heerenveen, The Netherlands) containing either 0 g or 250 mg cRG-I-extract filled with Maltodextrin (MALDEX 170, Tereos, Aalst, Belgium), with sunflower oil (0–10 mg) to aid encapsulation, and packed in blister strips (15 capsules per strip; 4 strips per pack). The test articles were identical for both treatments in capsule weight, size and colour, and outer packaging. Importantly, the maltodextrin used as placebo (DE 15.8) is a fully digestible carbohydrate absorbed in the small intestine and thus does not reach the colon to interact with the gut microbiota [34]. This ensured that the placebo had no confounding effects on colonic fermentation, allowing a valid assessment of the specific impact of cRG-I.

#### 2.1.3. Procedures

- *Anthropometric measurements*

Stature and body weight were measured at week 0 and week 4 utilising a precisely calibrated wall-mounted stadiometer and a levelled platform scale, respectively. All measurements were recorded with an accuracy of 0.1 cm for height and 0.1 kg for weight, ensuring participants were barefoot and dressed in lightweight attire to minimise extraneous variability. BMI was subsequently computed using the standard formula: weight in kilograms divided by the square of height in metres (kg*m^−2^).

- *Faecal samples*

Participants were provided with stool collection tubes (Sarstedt AB, Helsingborg, Sweden), equipped with spoon lid attachments, for at-home faecal sampling and were instructed to collect the samples using the EasySampler Stool Coll Europe kit (GP Medical Services, Hostelbro, Denmark) within a 48 h window preceding their weekly visit to the School of Medical Sciences at Örebro University at week 1, 2, 3 and 4. Participants were advised to immediately freeze the samples at −20 °C, after transporting them in an insulated container with ice packs (Sarstedt AB, Helsingborg, Sweden). Upon arrival at the facility, samples were transferred directly to −80 °C for long-term storage without further aliquoting.

- *Targeted qPCR*

Genomic DNA was extracted from 200 mg of frozen stool samples using the QIAamp PowerFecal Pro DNA Kit (QIAGEN GmbH, Stockholm, Sweden) and processed via the automated QIAcube^®^ Connect platform (QIAGEN GmbH, Sweden), strictly adhering to the manufacturer’s guidelines. DNA quality and purity were assessed using the Thermo Scientific Nanodrop 2000/2000C (Thero Fisher Scientific, Stockholm, Sweden) spectrophotometer to ensure reliable downstream analysis.

*Bifidobacterium* spp. quantification was performed using quantitative real-time PCR (qPCR) based on SYBR Green I fluorescence. A six-point standard curve, constructed from 10-fold serial dilutions of a reference strain provided by ProDigest (Ghent, Belgium), served as a benchmark for precise quantification. qPCR reactions were prepared in duplicates, with a total reaction volume of 12 µL, comprising 5 µL of 100× diluted DNA template, 10 µM of forward (5′-TCGCGTCYGGTGTGAAAG-3′) and reverse (5′-CCACATCCAGCRTCCAC-3′) primers (Eurofins Genomics, Ebersberg, Germany, Y = C/T, R = A/G), and the SensiMix™ SYBR^®^ Low-ROX Kit (Meridian Bioscience, Solna, Sweden). Thermal cycling conditions included an initial enzyme activation at 95 °C for 10 min, followed by 40 cycles of denaturation at 95 °C for 15 s, annealing at 60 °C for 30 s and elongation at 72 °C for 30 s. Melting curve analysis was conducted post-amplification to confirm product specificity, involving gradual cooling from 95 °C to 60 °C for one min and subsequent reheating to 95 °C. Amplification and data acquisition were performed on a Bio-Rad CFX96 Real-Time System C1600 Touch Thermal Cycler (Bio-Rad, Solna, Sweden), with data visualisation and storage managed using Bio-Rad CFX Manager software (version 3.1, 2012). The results were expressed as log *Bifidobacterium* copies/μL of extracted DNA solution, normalised to a DNA concentration of 100 ng/μL.

- *Faecal SCFA and BCFA analysis*

SCFAs, i.e., acetate, propionate, butyrate, and branched short-chain fatty acids (BCFAs) isobutyrate, isovalerate, and isocaproate were extracted from the faecal samples (0.1 g) using 1 mL water and 1 mL acetonitrile (with 0.5% 2-methyl hexanoic acid added as an internal standard). The extracts were analysed by capillary gas chromatography coupled with a flame ionisation detector, as described by De Weirdt et al. [35]. The system used was a GC-2014 gas chromatograph (Shimadzu, The Netherlands), equipped with a fatty acid-free EC-1000Econo-Cap capillary column (dimensions: 25 mm × 0.53 mm, film thickness 1.2 μm, Alltech, Belgium). The analysis was performed with a split injector, using a 1 mL injection volume, and the temperature was programmed to increase from 110 °C to 160 °C at a rate of 6 °C per min. Nitrogen was used as the carrier gas, and the injector and detector temperatures were set at 100 °C and 220 °C, respectively. Each measurement was conducted in single repetition for the assays.

- *Metabolic fingerprinting using laser-assisted rapid evaporative ionisation mass spectrometry (LA-REIMS)*

Samples were thawed at 4 °C and briefly vortexed (20 °C, 1 min). Approximately 100 µL of each sample was transferred to a 96-well plate and subjected to LA-REIMS analysis. The LA-REIMS platform used a MID infrared laser system (OpoletteTM HE2940, OPOTEK, LLC, Carlsbad, CA, USA) that consisted of a Q-switched Nd:YAG laser pumping Optical Parametric Oscillator (OPO). Transmission of the laser energy (wavelength of 2.94 µm, 175 µs pulse time) into the sample was achieved through free space optics, including a series of metallic-coated mirrors (OptoSigma Global Top, Les Ulis, France) and a Plano-convex lens (Thorlabs, GmbH, Bergkirchen, Germany). Mass analysis was carried out by means of a Xevo G2-XS Quadrupole Time-of-Flight (QToF) mass spectrometer (Waters Corporation, Wilmslow, UK), being operated in negative ionisation mode and applying an m/z scan range from 50 to 1200 Da. The main parameters concerned a cone and heater bias voltage of 50 V, a scan time of 0.3 s, and an isopropanol flow of 0.25 mL min^−1^ [36].

- *Inflammation marker*

After thawing faecal specimens from week 0 and 4, calprotectin (F-cal) concentrations were quantified by Unilabs (Skövde, Sweden) using a chemiluminescent immunoassay (CLIA) with a sandwich method approach [37]. Sample extraction was performed with the Liaison Calprotectin Stool Extraction Device using the weigh method for preparation and analyses were conducted on the Liaison XL Analyser. The detection threshold for the assay was established at 5 mg/kg, with values below this sensitivity limit categorised as undetectable and attributed a value of “zero”.

- *Blood samples and PBMC isolation*

Venous blood samples (8 mL) were collected after a minimum of 12 h of overnight fasting at week 0 and 4. The samples were drawn into BD Vacutainer^®^ CPT™ Tubes (Becton Dickinson, Franklin Lakes, NJ, USA) containing sodium heparin, and peripheral blood mononuclear cells (PBMCs) were subsequently isolated as per the manufacturer’s protocol. After centrifugation at 1600× *g* for 15 min at room temperature and two washes with phosphate-buffered saline (PBS; Gibco Thermo Fisher Scientific, Stockholm, Sweden), live cell counts were determined by trypan blue exclusion (Sigma-Aldrich, St. Louis, MO, USA) using a Bürker chamber. A total of 0.5 million cells were allocated to the unstained tube, while 5 million cells each were used for the phenotyping and fluorescence-minus-one (FMO) control tubes. Residual cells were cryopreserved in foetal bovine serum (FBS, Gibco Thermo Fisher Scientific, Stockholm, Sweden) supplemented with 10% dimethyl sulfoxide (DMSO) at −80 °C overnight before transferring to liquid nitrogen for long-term storage at a concentration of 5 million cells per tube.

- *FACS analysis*

Red blood cells were lysed with BD Pharm Lyse™ (BD Biosciences, San Jose, CA, USA) and washed with PBS before the Live/Dead staining. After lysis, cells were counted using a Bürker chamber. Immune cells were stained with Aqua fluorescent reactive dye (Invitrogen, Thermo Fisher Scientific, Waltham, MA, USA) for 5 min in the dark at room temperature, followed by Fc receptor blockade using FcR Blocking Reagent (Miltenyi Biotec, Bergisch Gladbach, Germany) under similar conditions. Cells were then incubated for 20 min at 4 °C with a panel of fluorescently conjugated monoclonal antibodies targeting CD66 (Miltenyi), CD86, CD123, CD3 (BD), HLA-DR, CD304, CD11c, CD14, CD16, CD19, CD20, and CD56 (BioLegend, San Diego, CA, USA) (Table S1). Cells were then rinsed with PBS to remove unbound antibodies. Cells were suspended in PBS for FACS analysis. Data were collected by Gallios flow cytometer (Beckman Coulter, Brea, CA, USA), analysed by Kaluza V2.2.1 software (Beckman Coulter, Indianapolis, IN, USA), and gating was performed as described in Figure S1. The proportions of plasmacytoid dendritic cells (pDC), myeloid DC (mDC) and monocytes were evaluated. The expression levels of the geometric mean fluorescent intensities (MFI) of the cellular markers were measured. Control samples were included for validation, comprising an unstained negative control, FMO controls lacking CD86 and CD304, and reference samples. The reference samples consisted of PBMCs isolated from the buffy coat of a single donor, stored in aliquots, and thawed immediately before analysis.

- *Questionnaires*

Validated questionnaires were employed to evaluate participants’ dietary fibre consumption, gastrointestinal symptomatology, physical activity, and quality of life.

Food Frequency Questionnaire (FFQ): A computational algorithm, developed by a dietitian at Örebro University (data not presented), processed data from a population-specific, standardised FFQ. The algorithm combined the FFQ data with dietary information obtained from three 24 h recalls (two weekdays and one weekend day) to generate a comprehensive dietary assessment [38]. This algorithm was employed during pre-screening to estimate average fibre consumption, which was used to stratify participants across the intervention groups.

The 15-item Gastrointestinal Symptom Rating Scale (GSRS) (AstraZeneca, Gothenburg, Sweden) was employed [39] at week 0 and 4. Participants provided self-reported ratings of the severity of gastrointestinal symptoms, including abdominal pain, indigestion, reflux, constipation, and diarrhoea. The severity of each symptom was rated on a scale from 1 to 7, where “0” indicated no symptoms and “7” indicated the most severe manifestation of the symptom.

Physical activity levels were evaluated using the self-reported International Physical Activity Questionnaire Short Form (IPAQ-SF) [40] at week 0 and 4. The IPAQ comprises four distinct domains: the first three domains assess the frequency and intensity of daily physical activity, expressed in metabolic equivalent of task (MET min per week), while the fourth domain captures the duration of sedentary behaviours.

At week 0 and 4, participants provided self-assessments of their general health status through the EQ-5D-5L instrument (EuroQol Research Foundation, Rotterdam, The Netherlands), a five-domain Likert-type scale questionnaire (scale from “no symptoms” to “severe symptoms”) encompassing mobility, self-care, routine activities, pain/discomfort, and anxiety/depression [41]. Additionally, participants evaluated their perceived overall health using a visual analogue scale ranging from 0 to 100, where “0” denoted the poorest conceivable health state and “100” signified optimal health.

#### 2.1.4. Outcomes

The primary outcome measures were changes in *Bifidobacterium* spp. abundance, and number of people with increased bifidobacteria counts. Statistically significant effects of cRG-I on the primary outcome parameters were considered measures of efficacy. Analyses that were not prespecified as primary outcomes were considered exploratory.

### 2.2. Preclinical Biological Function Assays

#### Gut Barrier Function

- *Colonic biopsies*

Sigmoid colonic biopsies were obtained following an overnight fast, with no bowel preparation to preserve the native mucosal environment, from ten healthy donors with a mean age of 35.7 years (range 20–63 years, not from the dietary intervention study but recruited using the same inclusion and exclusion criteria). Biopsies were collected using atraumatic forceps and immediately transferred into oxygenated Krebs–Ringer bicarbonate buffer. They were processed within 10 min and mounted in Ussing chambers (Harvard Apparatus Inc., Holliston, MA, USA) [42], which maintained their structural integrity and viability under constant oxygenation and temperature (37 °C). Biopsies were treated with 0.5 mg/mL cRG-I (unfermented), 2% *v/v* cRG-I (fermented), in the absence or presence of 1 mM sodium deoxycholate (SDC), a bile acid stressor known to disrupt epithelial barrier function as described earlier [43].

Transepithelial resistance (TER) reflecting the resistance across the full intestinal barrier, including both epithelial cells and underlying tissue layers, was used in this study to monitor biopsy viability. To establish a reliable baseline for each donor and ensure consistency among samples, median transepithelial resistance (MTER) was measured 20 min before treatment application. Biopsies were classified as non-viable and excluded if they exhibited a marked decline in TER, alongside irregular potential difference (PD) fluctuations that consistently fell outside the physiological range established by the donor’s other samples. Since baseline variability exists among individuals, viability was assessed within the context of each donor’s reference values to ensure reliable data inclusion.

Functional analyses assessed paracellular permeability via FITC-dextran flux and transcellular permeability via horseradish peroxidase (HRP) transport, with fluorescence and ELISA quantifications [43]. The transport of FITC-dextran and HRP was quantified as the difference between values at 90 min and baseline (ΔT_90_ − T_0_). Subsequent analyses were restricted to biopsies identified as SDC responders, defined as biopsies with a minimum 5% increase in paracellular (*n* = 7) or transcellular (*n* = 10) permeability following SDC treatment relative to untreated controls. All research activities involving human participants received ethical clearance from the Swedish Ethical Review Authority in Uppsala (Approval Number: Dnr. 2021-05142). The study was conducted in full compliance with the ethical standards outlined in the Declaration of Helsinki, and all participants provided written informed consent before engaging in the research.

Faecal samples from the same healthy adult biopsy donors, were collected earlier and in vitro static batch culture fermentations were conducted based on established protocols [44] omitting pH buffering. The aim of coupling faecal material to colonic biopsies from the same donor was to ensure a methodologically robust approach. Faecal inocula were prepared under anaerobic conditions and combined with sterilised growth medium containing key nutrients and cRG-I (1% *w/v*) as the test substrate, alongside negative and positive controls (basal medium without carbohydrate and inulin (#I2255, Sigma), respectively. Cultures were incubated at 37 °C for 6 h, the supernatant collected at 4 h was deemed optimal for generating biologically active metabolites and used in the Ussing chamber experiments (fermented cRG-I).

- *Caco-2/immune cell co-culture challenge model*

Short-term colonic batch fermentations were performed by ProDigest (Ghent, Belgium) as described previously [29,45], and cRG-I was dosed at 4 g/L. Supernatants were collected from the fermentation at 48 h for subsequent use in the co-culture experiment. Caco-2/immune cell challenge experiments were performed as described earlier [46]. Briefly, Caco-2 cells were maintained in Dulbecco’s Modified Eagle Medium (DMEM), supplemented with 10 mM HEPES and 20% heat-inactivated (HI) foetal bovine serum (FBS), while THP-1 cells were cultured in Roswell Park Memorial Institute (RPMI)1640 medium, supplemented with 10 mM HEPES, 1 mM sodium pyruvate, and 10% HI-FBS. Caco-2 cells were seeded at 1 × 10^5^ cells/insert and cultured for 14 days on semi-permeable inserts (cellQart) as described [45]. A functional monolayer was confirmed by measuring transepithelial electrical resistance (TEER, Millicell ERS-2) at the start of the co-cultures, reflecting the resistance across a uniform layer of intestinal epithelial cells in vitro. THP-1 cells (5 × 10^5^ cells/mL) were differentiated in 24-well plates with phorbol 12-myristate 13 acetic acid (PMA, 100 ng/mL) into macrophage-like cells for 48 h, as described [45]. After PMA was removed and cells were washed with PBS, the differentiated Caco-2 inserts were placed on top of the THP-1 cells. At the start of the co-culturing, fermented cRG-I (supernatants were filter-sterilised (0.22 µm) and diluted 1/5 in culture medium) was added to the Caco-2 cell layer on the apical side, and barrier integrity was assessed by measuring TEER again after 24 h incubation. Blank control medium from the colonic batch fermentations was included. Also, supernatant/medium from control Caco-2/THP-1 cells was included. All 24 h values were normalised to their own start value, after subtraction of the empty insert value, and are presented as a percentage of initial value (set at 100%). All experiments were performed in triplicate.

### 2.3. Statistical Analyses

- *Dietary intervention study:*

The group size for this study was calculated by referring to a previous dietary intervention study [27], which reported that 64% of participants receiving 300 mg/day of cRG-I exhibited an increase in *Bifidobacterium* spp., compared to 46% in the control group [7]. Given the higher cRG-I dosage (500 mg/day) and the additional stratification based on baseline bifidobacterial abundance, a more pronounced effect was anticipated in the present study. A power calculation using ‘the chance of a specific outcome’ for placebo versus cRG-I and the knowledge that we would determine bifidobacteria levels weekly (four observations per person) revealed that this study design could achieve approximately 78% power. The minimum number of participants required to detect the expected effect was calculated to be 22 per group. Anticipating a drop-out rate of 10% the study targeted an enrolment of 25 participants per group.

The results of continuous parameters were analysed via a General Estimating Equation (GEE) procedure in a Gaussian mode applying a stepwise approach. The change value per person per time interval per respective parameter was used as a dependent parameter. Continuous variables were characterised by their mean values ± standard deviation (SD) or standard error of the mean (SEM), as indicated, and categorical variables are reported as frequencies (n, %). The continuous parameters were bifidobacteria counts, change in number of people with increased bifidobacteria counts, concentration of microbial metabolites (SCFAs, BCFAs), faecal calprotectin levels, expression of immune activation markers (Δ %). Questionnaire-derived data comprised the continuous IPAQ-SF score, the EQ-5D-5L index (continuous), and ordinal scores from GSRS and EQ-5D-5L dimensions. Various confounding factors were considered in the analysis of the change data: start value of the relevant parameter, age, sex, BMI and fibre intake. Time was modelled in two ways: (1) as a weekly factor (when applicable) and (2) as an interaction term between time and the investigational product, with the latter coded as a dummy variable (0 = placebo, 1 = cRG-I) to account for time-dependent changes. The fit of the model was analysed via Wald chi square testing. Binary factors were addressed via logistic regression models to acknowledge their binomial character. Fit of the logistic model was analysed via Likelihood ratio chi square. Only when the model showed a significant explanation of the variation in the dependent parameter of the various explaining factors, showing a *p*-value below 0.05, were they regarded as statistically significant.

The Mann–Whitney U or chi-squared test was applied when comparing placebo to cRG-I for continuous and categorical parameters, respectively, assessed at baseline. The Wilcoxon signed-rank test was applied for assessing the effect of each treatment at week 4 compared to baseline. Statistical analyses were conducted for the intention-to-treat (ITT) population and per-protocol (PP) population. The presence of outliers was tested by applying the Grubbs’ test, which did not identify any significant outliers across the datasets. Therefore, all data points were retained for analysis. The outcome for the PP population was similar to that for ITT, unless indicated otherwise. Throughout the study, a *p*-value of 0.05 was considered to identify significance, applying two-sided evaluation based on the expected outcome of the parameters analysed. Statistical analysis was performed via Stata, version 12 (Statacorp, College Station, TX, USA) and GraphPad, version 6 (GraphPad Prism, San Diego, CA, USA).

LA-REIMS data were processed by performing log-transformation to induce normal distributions and unit variance scaling (1/standard deviation [SD]) to standardise the range of signal intensities; then, the normalised data were subjected to multivariate statistical analysis using SIMCA 17 (Sartorius, Göttingen, Germany). Unsupervised Principal Component Analysis (PCA-X) was executed to assess the natural patterning of samples and reveal potential outliers (based on the Hoteling’s T2 criterion). Orthogonal Partial Least Squares Discriminant Analysis (OPLS-DA) was used to differentiate samples according to the experimental conditions in a supervised fashion. The validity of the OPLS-DA models was verified by permutation testing (n = 100), cross-validated analysis of variance (*p*-value < 0.05), and the quality parameter Q2(Y) (≥0.5). In general, the model performance is described by R2(X) (the predictive and orthogonal variation in X-values, i.e., m/z features), R2(Y) (the ability to predict the Y-data for the specifically used dataset, i.e., predicting the sample classification), and Q2 (the ability to correctly predict the Y-data when an external dataset would be considered).

- *Pre-clinical studies:*

In the ex vivo Ussing chamber and in vitro Caco-2/THP-1 experimental models, electrophysiological and permeability parameters—including TER, FITC-dextran, and HRP transport—were analysed by averaging biological replicates for each condition. Comparisons between control and treatment groups were conducted using the Wilcoxon signed-rank test, applied to both Ussing chamber-derived permeability data and TEER measurements in the Caco-2/THP-1 co-culture across donors. Statistical computations were executed using IBM^®^ SPSS^®^ Statistics version 28, with a predefined significance threshold of *p* < 0.05.

## 3. Results

### 3.1. Human Dietary Intervention—Study Subjects

In total, 143 of the 257 participants invited were screened, of whom 54 were enrolled in the study between September 2023 and March 2024, with 28 in the placebo group and 26 in the cRG-I group (Figure 1A,B). Of the 54 randomised participants, 2 participants in the cRG-I group dropped out due to the use of antibiotics at the start and after one week of the supplementation period, for reasons unrelated to the study. The characteristics of the participants are presented in Table 1, which shows comparable attributes between the placebo and cRG-I supplementation groups. No statistically significant differences were found between the groups for the characteristics measured. BMI values were in the slightly overweight range for both groups (placebo: 26.20 ± 3.45; cRG-I: 24.71 ± 3.46, *p* = 0.200). Participant sub-group allocation was determined using baseline bifidobacteria counts and daily fibre intake (Figure S2). Overall compliance in terms of the daily intake of the test article and adherence to the protocol were excellent (placebo: 95.7%, cRG-I: 94.4%), with a successful stratification and randomisation procedure.

### 3.2. Safety and Tolerability

The intake of the test articles for 4 weeks did not lead to adverse events, and no participants withdrew due to adverse reactions associated with either the placebo or cRG-I. Furthermore, the tested products were well-tolerated and had no notable impact on participants’ self-reported intestinal health or overall well-being.

### 3.3. Effect of cRG-I Supplementation on Numbers of Bifidobacteria

**Figure 2:**
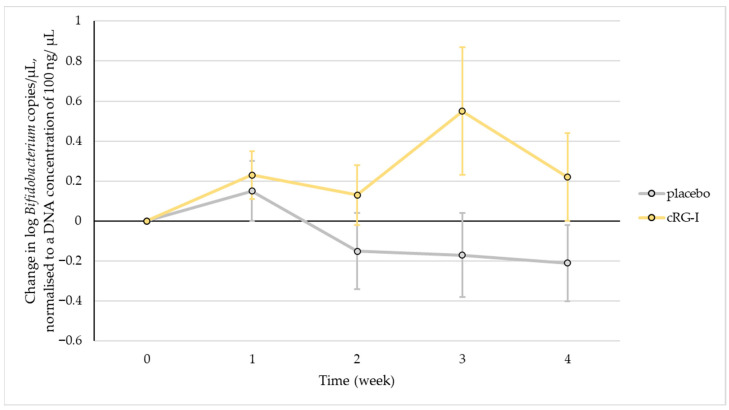
The change in the bifidobacteria counts during 4 weeks after the start of supplementation. Each symbol depicts the mean ± SEM of at least 22 observations. Significance is not indicated on the figure, as changes over time were analysed using GEE and are described in the results.

In this study, 500 mg/day cRG-I was supplemented every day for 4 weeks and bifidobacteria counts were assessed every week in healthy subjects that had a diet with a relatively high fibre content (approximately 25 g/d in the placebo group and 28 g/d in the cRG-I group). A significant increase in the change in bifidobacteria counts over time was observed in the cRG-I group *p* = 0.017 (Figure 2), and the distribution of individual responses is shown in Supplementary Figures S3 and S4. In the first week, the change in bifidobacteria counts was similar for both groups but increased gradually in the cRG-I group throughout the study, with levels peaking at week 3 in a 1.5-fold increase compared to baseline. The percentage of subjects with an increase in bifidobacteria counts was higher in the cRG-I group at all time points compared to the control group.

Specifically, the change in the % of subjects with an increased bifidobacteria count decreased in both groups between week 1 and 2; this continued to decrease in the placebo group for the subsequent 3 weeks and then increased between week 2 and 3 for the cRG-I group, and then finally decreased between week 3 and 4 (Figure S5). At week 4, 43% of subjects in the placebo group and 57% of subjects in the cRG-I group had increased bifidobacteria counts.

### 3.4. Effect of cRG-I on Production of Microbial Metabolites

SCFAs, mainly acetate, propionate, and butyrate, are primarily generated in the colon through fermentation of non-digestible dietary fibre by the gut microbiota. Dietary fibre intake was comparable at the start of the intervention between the groups (Table 1), and participants were instructed to maintain their dietary habits throughout the study. The only statistically significant difference observed in faecal SCFA levels was for isobutyric acid, which was higher in the cRG-I group after 4 weeks (5.96 ± 1.04 vs. 6.75 ± 1.91, *p* = 0.034, Table S2). No differences in the levels of the other SCFAs in faecal samples were observed (Table S2). Finally, LA-REIMS analysis was performed to assess whether metabolic fingerprints in faecal samples differed before and after the 4-week dietary supplementation. Multivariate analysis of the LA-REIMS data did not indicate any treatment-related effects (Figure S6).

### 3.5. Effect of cRG-I on Health Parameters

**Figure 3:**
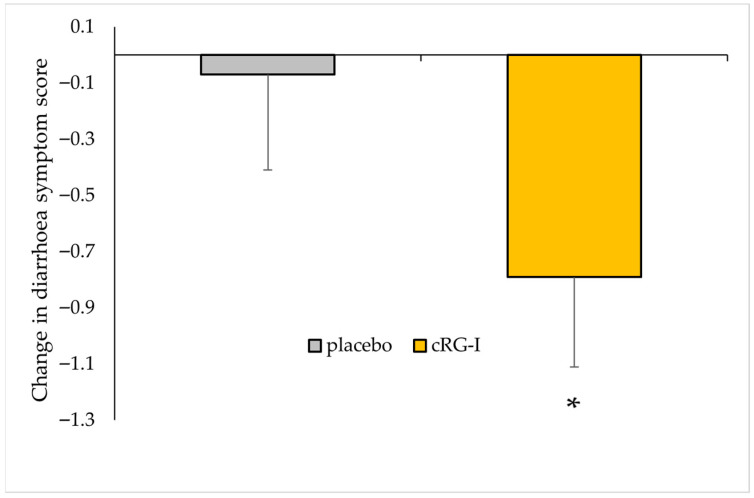
The change in diarrhoea score between baseline and week 4 in healthy subjects was larger in the cRG-I group compared to the placebo. *: statistically significant compared to placebo (*p* < 0.05).

The effect of the dietary intervention on a variety of health parameters was assessed before and after 4 weeks using validated questionnaires. Gut health parameters were assessed using the GSRS, which included questions on total gastro-intestinal symptoms, abdominal pain, reflux, indigestion, diarrhoea and constipation. The diarrhoea score was the only parameter that demonstrated a statistically significant change (Figure 3), with 4.50 ± 1.94 at week 0 and a reduction to 3.67 ± 1.2 after the intervention with cRG-I (*p* < 0.04). The change depicted in Figure 3 represents the difference between week 4 and baseline values, with a negative change indicating an improvement (i.e., firmer stool consistency). Among participants receiving cRG-I, 8 out of 26 reported a lower diarrhoea score at the end of the intervention period, compared to 7 out of 28 in the placebo group. Since all participants were healthy throughout this study, the reduction in diarrhoea score should be interpreted as increased firmness of stool material after 4 weeks rather than a reduction in the manifestation of diarrhoea (max score of 7). Furthermore, no differences in faecal calprotectin levels were observed between the groups.

Physical activity was assessed using IPAQ. No changes in MET-min/week were reported for total physical activity or walking. Vigorous activity levels were maintained in the cRG-I group, with a mean of 672.92 ± 251.49 MET-min/week at baseline and 668.33 ± 302.08 MET-min/week after 4 weeks (mean ± SEM). In contrast, the placebo group showed a reduction from 1001.43 ± 184.70 to 699.26 ± 172.38 MET-min/week. When comparing the groups, the change was statistically significant (*p* < 0.04). Self-reported moderate activity levels decreased in the cRG-I group from 1173.08 ± 279.78 to 776.67 ± 153.37 MET-min/week, while they increased in the placebo group from 645.71 ± 131.15 to 910.71 ± 165.86 MET-min/week. The between-group difference in change was significantly lower in the cRG-I group (*p* < 0.04). Together, these results show distinct patterns of change in activity levels between the two groups over the intervention period.

Quality of life was assessed using QoL EQ-5D-5L. Health perception did not change in time or by the intervention, and no differences were observed between the groups for any of the reported parameters.

### 3.6. Effects of cRG-I on Immune Cell Activation

**Figure 4:**
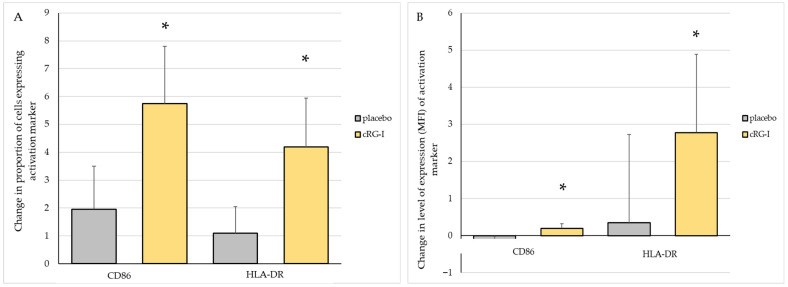
(**A**) Change in the proportion of cells expressing activation markers and (**B**) change in expression of cell surface activation markers (MFI) on human blood dendritic cells (mDCs) after 4 weeks intervention. Data are baseline-corrected and presented as mean ± SEM (%) at week 4. *: statistically significant compared to placebo over time (*p* < 0.05).

The dietary intervention did not affect the proportions of the pDCs, mDCs or monocyte cell populations. The activation status of pDCs, mDCs and monocytes was assessed using a flow cytometer by measuring the % cells expressing CD86 and HLA-DR as well as the mean fluorescence intensity (MFI) of these cell surface markers at week 0 and week 4. The proportion of mDCs expressing CD86 and HLA-DR increased over time in both the cRG-I and placebo groups. After 4 weeks, in the cRG-I group, the change was significantly higher for the proportion of mDCs expressing CD86 and HLA-DR (*p* < 0.005) compared to the placebo group (Figure 4A). Also, in the cRG-I group, the CD86 mean fluorescence intensity (MFI) significantly increased in time (*p* < 0.02), as did the expression of HLA-DR on the cell surface of mDCs (*p* < 0.05) compared to the placebo group (Figure 4B).

For pDCs, no difference was observed between treatment groups in the proportion of cells expressing CD86, while the proportion of cells expressing HLA-DR in the cRG-I group tended to increase but did not reach statistical significance. Interestingly, CD86 expression tended to increase on the cell surface (MFI) of pDC in the cRG-I group while HLA-DR did not change. For monocytes, no differences were observed in the proportion of cells; however, cell surface expression tended to increase for both markers in the cRG-I group.

### 3.7. Preclinical Gut Barrier Function Assays Using In Vitro and Ex Vivo Challenge Models

To better understand the mechanisms behind the potential health benefits of cRG-I, this study also investigated the effects of fermented cRG-I (from 48 h batch fermentation) using in vitro models. Gut barrier function was studied using a Caco-2/THP-1 co-culture immune challenge model, and a challenge model with colonic biopsies in Ussing chambers.

#### 3.7.1. Effects of cRG-I in a Co-Culture Caco-2/THP-1 Challenge Model

**Figure 5:**
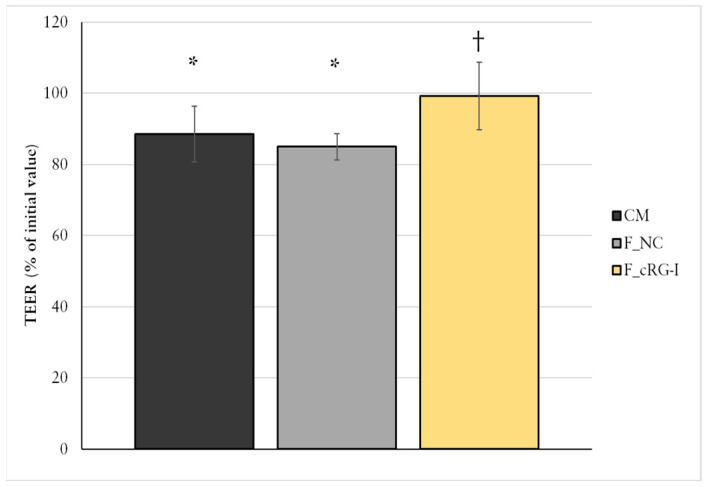
Protective effect of fermented cRG-I on gut barrier integrity assessed using an in vitro Caco-2/THP-1 co-culture model of epithelial inflammation. TEER values (% of initial) were measured 24 h after PMA stimulation. CM: cell culture medium; F_NC: faecal fermentation supernatant without carbon source; F_cRG-I: faecal fermentation supernatant with cRG-I. Bars represent mean ± SD of three technical replicates. * *p* < 0.05 compared to baseline of each treatment; † *p* < 0.05 compared to F_NC; (Wilcoxon matched-pairs signed-rank test).

Fermented cRG-I (F_cRG-I), which is a pool of microbial metabolites including SCFAs and fragments of cRG-I, revealed protective benefits on intestinal epithelial barrier integrity, as demonstrated in an inflammation-induced barrier disruption model using a co-culture of Caco-2 and THP-1 cells (Figure 5). After 24 h of apical exposure to the medium control, co-cultured THP-1-derived macrophage-like cells induced a significant reduction in TEER (i.e., barrier disruption) in the controls. In contrast, F_cRG-I, derived from 48 h colonic fermentation, preserved TEER levels, with values remaining similar to baseline values and significantly different to the control sample from the batch fermentation (*p* = 0.036).

#### 3.7.2. Effects of cRG-I in a Colonic Biopsy Challenge Model

Biopsies were either untreated or treated with cRG-I (0.5 mg/mL) or fermented cRG-I (F_cRG-I; 2% *v/v*) and challenged with SDC (1 mM), which is an intestinal barrier stressor. Biopsies challenged with SDC exhibited a lower TER value after 90 min than SDC-challenged biopsies treated with cRG-I (*p*_T90_ = 0.011) or F_cRG-I (*p*_T90_ = 0.005), suggesting a protective effect of the treatment.

**Figure 6:**
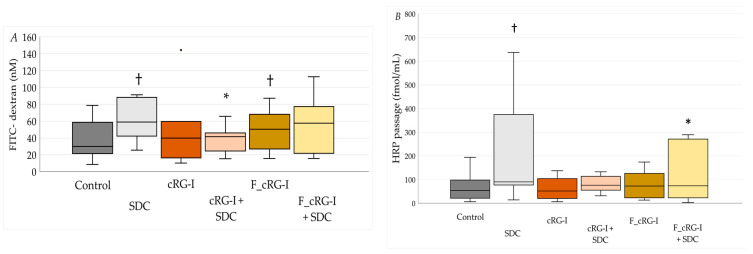
The effect of cRG-I on gut barrier integrity on colon biopsies. (**A**) Effects on colonic paracellular permeability (absolute concentration values) in biopsies mounted in Ussing chambers for SDC responders (control n = 7), SDC (n = 6), cRG-I (n = 7), cRG-I + SDC (n = 7), F_cRG-I (n = 7), F_cRG-I + SDC (n = 7). (**B**) Effects on transcellular permeability (absolute concentration values) in biopsies mounted in Ussing chambers for SDC responders (control n = 10), SDC (n = 9), cRG-I (n = 10), cRG-I + SDC (n = 10), F_cRG-I (n = 10), F_cRG-I + SDC (n = 10). † *p* < 0.05 statistically significant compared to control; * *p* < 0.05 statistically significant compared to SDC; Wilcoxon matched-pairs signed rank test.

Biopsies exhibited augmented levels of paracellular permeability when challenged with SDC after 90 min in the Ussing chamber compared to baseline (*p* = 0.028). Pre-treatment with cRG-I did not affect permeability when compared to control biopsies, whereas pre-treatment with fermented cRG-I (F-cRG-I) exhibited increased paracellular permeability levels (*p*_F_cRG-I_ = 0.028). Pre-treatment with cRG-I protected barrier function, i.e., no increase in paracellular permeability when biopsies were challenged with SDC. The increase in permeability induced by fermented cRG-I was not further increased by SDC challenge (Figure 6A).

Biopsies also exhibited increased levels of transcellular permeability when challenged with SDC after 90 min in the Ussing chamber compared to baseline (*p* = 0.008), while biopsies pre-treated with cRG-I or F_cRG-I displayed no change in transcellular permeability when compared to the control. When challenged with SDC, the biopsies that were pre-treated with cRG-I tended to have lower levels of transcellular permeability than the control biopsies that were untreated and challenged with SDC, but this did not reach statistical significance (*p*_cRG-I+SDC_ = 0.066). The biopsies pre-treated with F_cRG-I did not demonstrate a statistically significant increase in transcellular permeability levels when challenged with SDC (*p*_F_cRG-I+SDC_ = 0.028) (Figure 6B).

## 4. Discussion

This paper presents the outcome of the second randomised, double-blind, placebo-controlled study in healthy adults with cRG-I. This study was designed to extend the existing scientific evidence on the beneficial prebiotic effects of cRG-I and to investigate whether a shorter duration (4 weeks) of dietary supplementation, with a slightly higher dose (500 mg/day), would also have microbiota and immune modulatory effects. Our primary aim was to establish whether a bifidogenic effect could be achieved within 4 weeks, so we measured the change in abundance of *Bifidobacterium* spp. every week for 4 weeks. Secondary aims were to measure the effects on faecal SCFA and BCFA, and faecal calprotectin levels, and to assess the expression of activation markers on circulatory immune cells. We also employed a variety of validated questionnaires to explore potential effects on gut health and well-being. In addition, we used preclinical models to explore and better understand the potential beneficial effects of cRG-I on gut barrier integrity, hypothesising that cRG-I supplementation would result in the production of metabolites that might protect the gut barrier when challenged.

A key methodological advancement of this study was the stratification of participants based on baseline dietary fibre intake and bifidobacteria counts. Participants were categorised into treatment groups according to high or low bifidobacteria counts and high or low dietary fibre intake, allowing for a more precise evaluation of cRG-I’s effects. The mean fibre intake was 25 g/d in the placebo group and 28 g/d in the cRG-I group and surprisingly met the recommended daily intake of 25 g/day for adults [3]. Given that over 70% of participants in each group were women, these intake levels are notably high compared to the Nordic dietary average for women (16–22 g/day) [47]. Furthermore, while baseline fibre intake was used as a stratification factor to ensure balanced randomisation, the study was not powered for subgroup analyses based on dietary fibre levels. Future larger-scale intervention trials with greater sample sizes per group would be required to allow such sub-analyses and to further explore whether habitual dietary fibre intake influences the response to cRG-I supplementation.

Notably, despite the short intervention period, the high fibre intake combined with the low daily dose (500 mg/day), supplementation with the pectic polysaccharide cRG-I significantly increased faecal *Bifidobacterium* spp. levels in healthy adults. In an earlier intervention study that did not assess daily fibre intake, a bifidogenic effect of cRG-I was demonstrated after 8 weeks with daily doses of 300 mg and 1.5 g [7]. The present study therefore confirms and extends these findings by showing that significant microbiota modulation can be achieved within a shorter timeframe, while also providing a dynamic view of the temporal response through weekly assessments. The observed peak at week 3 suggests that even low-dose cRG-I exerts a gradual yet measurable enrichment of bifidobacteria with consecutive consumption, the timing of which could not have been predicted in advance. This highlights the value of frequent sampling to capture the short-term dynamics of the gut microbial response. While a low dose of cRG-I will only have a limited contribution to total dietary fibre intake, it is apparent that such a low dose can significantly modulate the gut microbiota composition and selectively increase the growth of bifidobacteria species, even in subjects with a relatively high intake of dietary fibre. The practical advantages for supplementation with a low-dose cRG-I include compliance and convenience, as it is well tolerated without reported gastrointestinal discomfort such as bloating or flatulence, which are common complaints with some prebiotic fibres. Additionally, low doses are easier to incorporate into food and beverage formulations and are especially suitable for dietary supplements with small dosage formats, thereby enhancing the applicability of cRG-I in functional nutrition solutions. The most established prebiotics are fibres with simple chemical structures, such as fructo-oligosaccharides (FOS), inulin, and galacto-oligosaccharides (GOS), that often require a daily dose of 2–15 g per day [48].

Furthermore, while the supplement was well tolerated, no effects were observed on self-reported overall health perception. Interestingly, when assessing lifestyle parameters, we noted a redistribution in physical activity patterns between vigorous and moderate intensity, without a net reduction in total activity levels. This shift was most pronounced in the placebo group, which may partly reflect seasonal influences since the study took place during the winter months in Sweden (October–March), a period generally associated with reduced opportunities for vigorous outdoor exercise. These fluctuations therefore likely represent natural lifestyle variation rather than an intervention effect.

A variety of health benefits have been attributed to *Bifidobacterium* species and include a healthy, resilient gut microbiota [49], the production of beneficial metabolites and vitamins, the growth of other beneficial gut bacteria via cross-feeding, the prevention of gut disorders, anti-pathogenic effects, and immune modulation [21,50,51,52,53]. Depletion of *Bifidobacterium* has been linked to inflammatory bowel diseases and irritable bowel syndrome [54]. The bifidogenic effect of cRG-I is most likely explained by its structural characteristics, particularly the RG-I backbone with arabinose- and galactose-rich side chains that are selectively utilised by *Bifidobacterium* spp. In colonic fermentation models, cRG-I has been shown to consistently stimulate the growth of *B. longum* and *B. adolescentis* [29], while mucosal-simulator of human intestinal microbial ecosystem (M-SHIME) experiments further demonstrated that *B. longum* can benefit from cross-feeding on oligosaccharides released from these side chains by primary degraders [30]. This structural–microbial interplay provides a mechanistic rationale for the bifidogenic response observed in the present intervention. In this study, bifidogenic effects peaked at 3 weeks, and while we hypothesised that fermentation of cRG-I would lead to increased SCFA production, this was not evident in faecal measurements. Despite some studies suggesting the potential usefulness of measuring SCFAs in faeces [55], Von Engelhardt et al. reported that more than 95% is absorbed in the large bowel [56]. Considering this, it was not surprising that no differences in the levels of SCFAs in faecal samples between placebo and cRG-I were evident, and only one significant difference was observed, in isobutyric acid levels in a limited number of subjects (n = 14). Nonetheless, in vitro and ex vivo studies using colonic batch fermentation models (SHIME^®^ and SIFR^®^ technology) have demonstrated increased SCFA production as a result of cRG-I fermentation [29,30,31,57]. The gut microbiota can produce a wide range of metabolites that can contribute to health locally, e.g., by maintaining gut barrier integrity and gut homeostasis, or systemically. Recent ex vivo data showed that fermentation of cRG-I also results in the production of tryptophan metabolites like indole-3-propionic acid (IPA) [32]. In this study, we used LA-REIMS technology to explore potential treatment effects on the metabolic fingerprints in faecal samples from healthy subjects before and after 4 weeks of dietary supplementation. The only differences that were observed, between the start and the end of the intervention in both the placebo and cRG-I groups, may be attributed to inter-individual donor variations or time effects. However, the absence of detectable effects in both untargeted and targeted analyses does not necessarily imply a lack of biological activity. It is possible that subtle or transient metabolic changes occurred that were not captured due to limitations in sensitivity, timing, the dose used, or the choice of analytical platform and sample matrix.

Our first clinical intervention study showed accelerated and enhanced protective innate immune and anti-viral interferon responses to a challenge with a common respiratory virus in healthy subjects taking only 300 mg/day of cRG-I per day [27], as well as activation of natural killer cells [28]. Based on these outcomes, we hypothesised that cRG-I modulates immune responsiveness by priming or training dendritic cells. Dendritic cells have been described as the first line of defence against invading pathogens, and they react to cell damage [58]. In this study, dietary supplementation with 500 mg/day cRG-I resulted in an immune modulatory effect. Specifically, cRG-I significantly increased the expression of activation markers on circulatory mDCs and tended to increase the expression of activation markers on pDCs, thereby supporting our hypothesis. In vitro investigations also showed that cRG-I increased the expression of activation markers on mDCs and pDCs isolated from buffy coats, showing that cRG-I can exert direct effects on these immune cells (manuscript in preparation). Further studies are needed to elucidate whether the activated DCs from the dietary intervention were activated through direct interactions with cRG-I as it passed through the small intestine. For example, are DCs in the Peyer’s patches sampling cRG-I in the luminal content and migrating via the blood to other locations. Alternatively, could DCs have been activated indirectly through systemic SCFA activation of G-protein coupled receptors (GPCRs) or inhibition of histone deacetylases, potentially leading to epigenetic changes and immune priming/training [59]. Other dietary interventions that have shown activation of DCs include bovine lactoferrin, which is also associated with reduced numbers of rhinovirus infections [60], and a heat-killed *Lactococcus lactis* strain [61,62].

Functional defects in the intestinal epithelial barrier appear to play a central role in the pathogenesis of inflammatory bowel disease, irritable bowel syndrome, sepsis and type-2 diabetes [63], suggesting that a protective effect on the intestinal barrier could be important for the prevention and management of such diseases. Preclinical data supports the potential benefits of cRG-I and its fermentation end-products on protecting gut barrier integrity [29]. Both cRG-I and fermented cRG-I preserved the viability of colonic tissues and protected the gut barrier function by maintaining colonic paracellular (cRG-I) and transcellular permeability (F_cRG-I) in the presence of challenge with SDC. Although SDC is employed to induce barrier dysfunction, it may not fully mimic the complexity of human disease conditions, and responses can vary considerably among individuals, even within a healthy population, with non-responders in some cases. Such variability may arise from differences in baseline gut barrier integrity, cellular sensitivity and microbiota composition. Nevertheless, the observed results are in line with other research highlighting the role of dietary polysaccharides in modulating gut barrier integrity. For instance, rhamnogalacturonan (RGal), a pectic polysaccharide from *Acmella oleracea*, has been shown to enhance intestinal barrier function in colonic epithelial cell models [64]. Similarly, lemon pectins with varying degrees of methyl esterification protected the epithelial barrier [65], and apple pectin, particularly its highly methylated form, improved gut barrier function by promoting the expression of tight junction proteins, such as claudin-1 and ZO-2, in the cecum of NOD mice [66]. Another study demonstrated that pectic polysaccharides from Goji berry (74% RG-I) and raspberry (62% RG-I), primarily composed of RG-I, effectively alleviated gut inflammation and enhanced intestinal barrier function in a DSS-induced IBD mouse model [67].

## 5. Conclusions

In conclusion, the results from this study, following a dietary intervention with 500 mg/day of cRG-I in subjects consuming a diet relatively high in fibre, are in line with the previously described dual mode of action [7,30,31]. Firstly, the selective enrichment of beneficial gut microorganisms is independent of the initial baseline microbiota composition, potentially reducing the risk of non-responders. Secondly, the increased expression of activation markers, on circulating dendritic cells, supports the notion that cRG-I primes or trains the (innate) immune system. In addition, the data suggest a third mode of action, namely that cRG-I has a beneficial effect on gut barrier function. The small improvement in diarrhoea score in the cRG-I group and the in vitro data supporting a potential beneficial effect on gut barrier function make it tempting to investigate the effects of cRG-I on health outcomes in challenged populations, e.g., in subjects with irritable bowel syndrome (IBS) or with inflammatory bowel disease (IBD), or even with metabolic disorders where the aetiology is associated with a compromised gut barrier or “leaky-gut”.

## Figures and Tables

**Table 1:** Baseline characteristics of subjects.

Parameter	Statistic	Placebo	cRG-I	*p*-Value
N		28	26	
Age (y)	Mean ± SD	48.4 ± 12.1	46.4 ± 17.0	*p* = 0.815
Female sex	N (%)	19 (67.9%)	21 (80.8%)	*p* = 0.279
BMI (kg*m^−2^)	Mean ± SD	26.20 ± 3.45	24.71 ± 3.46	*p* = 0.200
Bifidobacteria counts ^a^ at baseline	Mean ± SD	1.09 ± 0.89	1.00 ± 0.07 ^b^	*p* = 0.539
Energy (kcal/day)	Mean ± SD	1904.75 ± 644.69	2214.49 ± 915.88	*p* = 0.402
Protein (%/day)	Mean ± SD	18.62 ± 2.62	17.27 ± 2.93	*p* = 0.121
Carbohydrates (%/day)	Mean ± SD	39.08 ± 7.49	43.76 ± 7.55	*p* = 0.098
Fibre intake (%/day)	Mean ± SD	2.48 ± 0.84	2.52 ± 0.94	*p* = 0.972
Fibre intake (g/day)	Mean ± SD	25.25 ± 12.25	28.28 ± 20.94	*p* = 0.640
Fat (%/day)	Mean ± SD	38.40 ± 6.15	35.06 ± 6.10	*p* = 0.109
Dietary supplements consumption	N (%)	8 (28.6%)	9 (34.6%)	*p* = 0.633

^a^ Expressed in log *Bifidobacterium* copies/µL, normalised to a DNA concentration of 100 ng/µL. ^b^ N = 24 at baseline for this parameter due to drop-outs. Statistical analyses performed with Mann–Whitney U test or Chi-square.

## Data Availability

Due to ethical restrictions and privacy considerations regarding participant data, the data presented in this study are available on request from the corresponding author.

## Supplementary Materials

The following supporting information can be downloaded at: https://www.mdpi.com/article/10.3390/microorganisms13092156/s1, Figure S1. Gating strategy FACS. Forward and side scatter parameters were used to assess cell size and granularity; Figure S2. Participants sub-group allocation based on baseline bifidobacteria counts and daily fibre intake; Figure S3. Distribution of individual changes in *Bifidobacterium* counts during four weeks of placebo supplementation. Each dot represents one subject, and values are expressed as change in log *Bifidobacterium* copies/µL normalised to a DNA concentration of 100 ng/µL. Figure S4. Distribution of individual changes in *Bifidobacterium* counts during four weeks of cRG-I supplementation. Each dot represents one subject, and values are expressed as change in log *Bifidobacterium* copies/µL normalised to a DNA concentration of 100 ng/µL. Figure S5. The change in the percentage of persons with an increase in bifidobacteria counts (as compared to the value at the start) during 4 weeks after the start of the consumption of various compounds; Figure S6. PCA score plot illustrating the potential impact of cRG-I dietary supplementation on LA-REIMS-derived metabolic fingerprints in healthy subjects; Table S1. Antibodies used for surface staining in flow cytometry (FACS) analysis, including fluorochrome, clone, vendor, catalogue number, and volume per test; Table S2. Effects of cRG-I dietary supplementation on faecal SCFAs and BCFAs (absolute values (μmol/g faeces).
