# Supplementary material for: Carrot Rhamnogalacturonan-I Supplementation Shapes Gut Microbiota and Immune Responses: A Randomised Trial in Healthy Adults
Source: Microorganisms. 2025 Sep 16;13(9):2156. doi: 10.3390/microorganisms13092156 (PMC12472267; doi:10.3390/microorganisms13092156)
Supplement: Supplementary file 1 [file microorganisms-13-02156-s001.zip › microorganisms-3820620-supplementary.pdf]

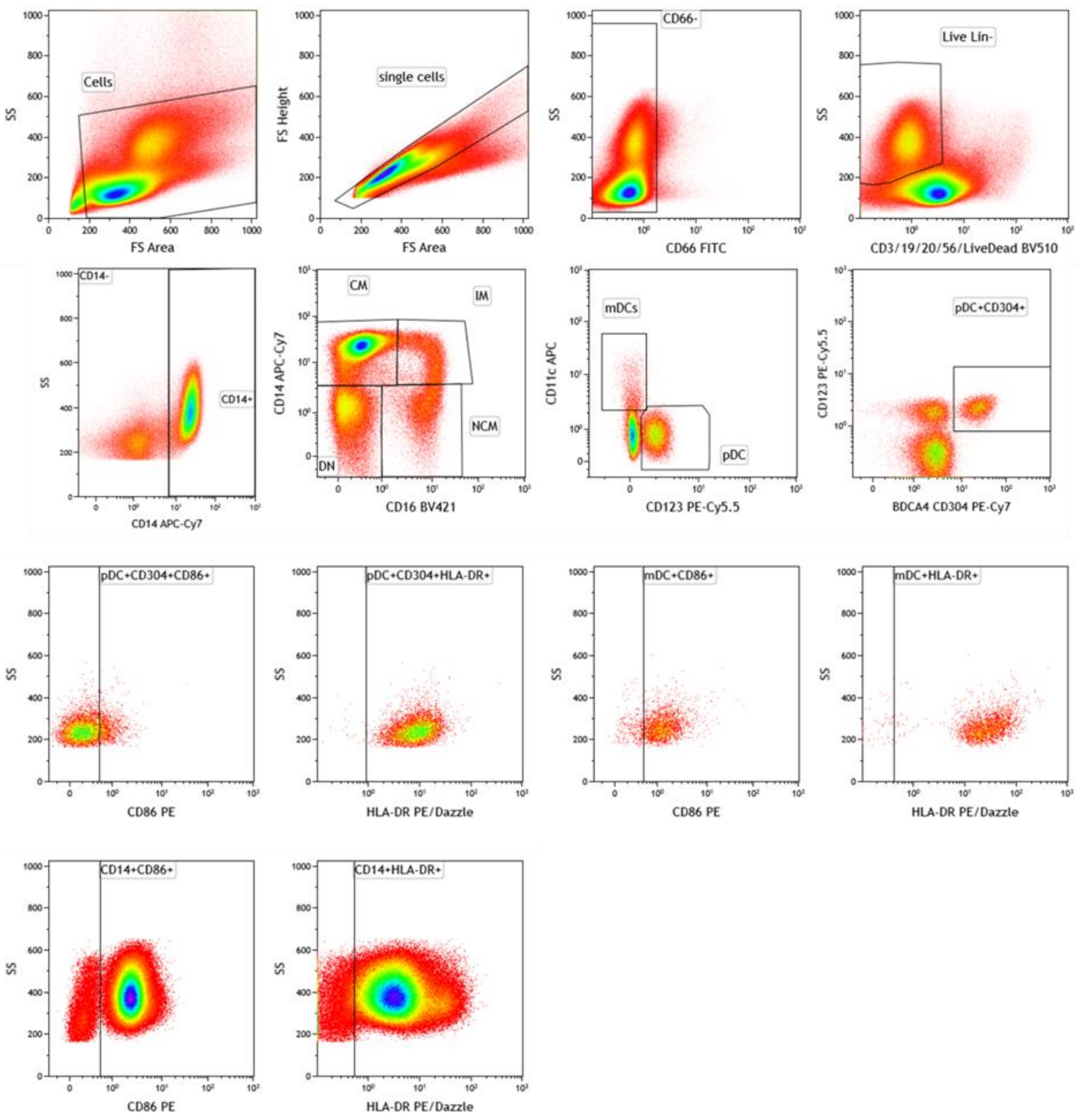

Figure S1. Gating strategy FACS. Forward and side scatter parameters were used to assess cell size and granularity. Single cells negative for neutrophils ($CD66^{-}$) were further gated on live cells based on both Live/Dead and Lineage (CD3, CD19, CD20, CD56) staining. Live cells were then categorised into subsets based on CD14 and CD16 expression: classical monocytes ($CD14^{+}$), intermediate monocytes ($CD14^{+}CD16^{+}$), non-classical monocytes ($CD16^{+}$), and double-negative monocytes ($CD14^{-}CD16^{-}$). The double-negative population was further characterised using $CD11c^{+}$ (mDCs) and $CD123^{+}$ $CD304^{+}$ (pDCs). mDCs, pDCs, and classical monocytes were subsequently analysed for CD86 and HLA-DR expression. Gating was based on the unstained negative control and FMO controls lacking CD86 and CD304.

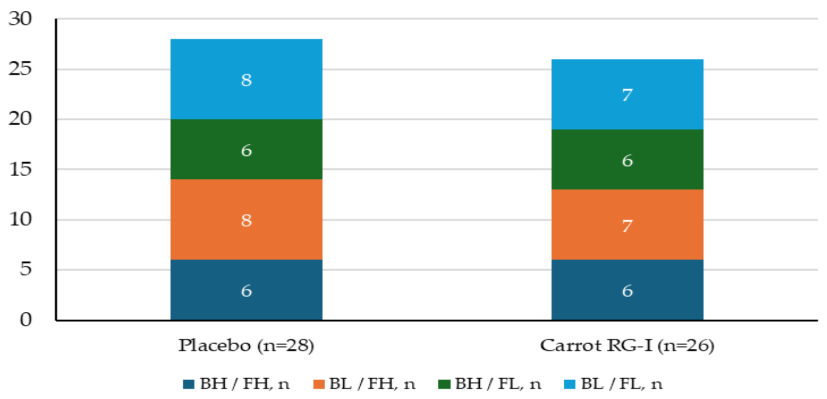

Figure S2. Participants sub-group allocation based on baseline bifidobacteria counts and daily fibre intake. BH/ BL: High or Low bifidobacteria counts at baseline compared to the bifidobacteria levels measured in participants using the median value obtained from group A enrolled in the study (log *Bifidobacterium* copies/µl, normalised for DNA concentration of 100 ng/µl ≥ or < 0.894). FH/ FL: High or Low daily dietary fibre intake at baseline compared to the daily dietary fibre intake of participants using the median value obtained from group A enrolled in the study (daily dietary fibre intake ≥ or < 22.66 g/day).

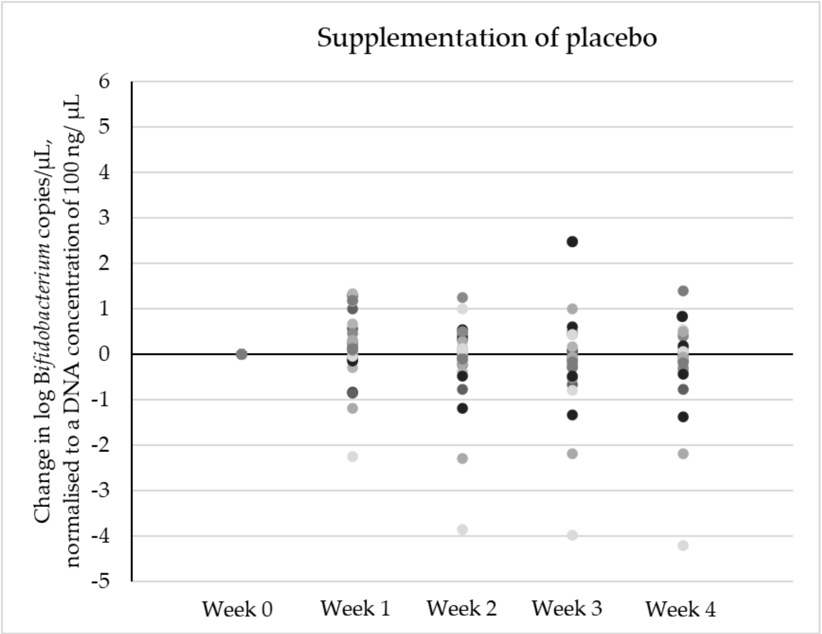

Figure S3. Distribution of individual changes in *Bifidobacterium* counts during four weeks of placebo supplementation. Each dot represents one subject, and values are expressed as change in log *Bifidobacterium* copies/µL normalised to a DNA concentration of 100 ng/µL.

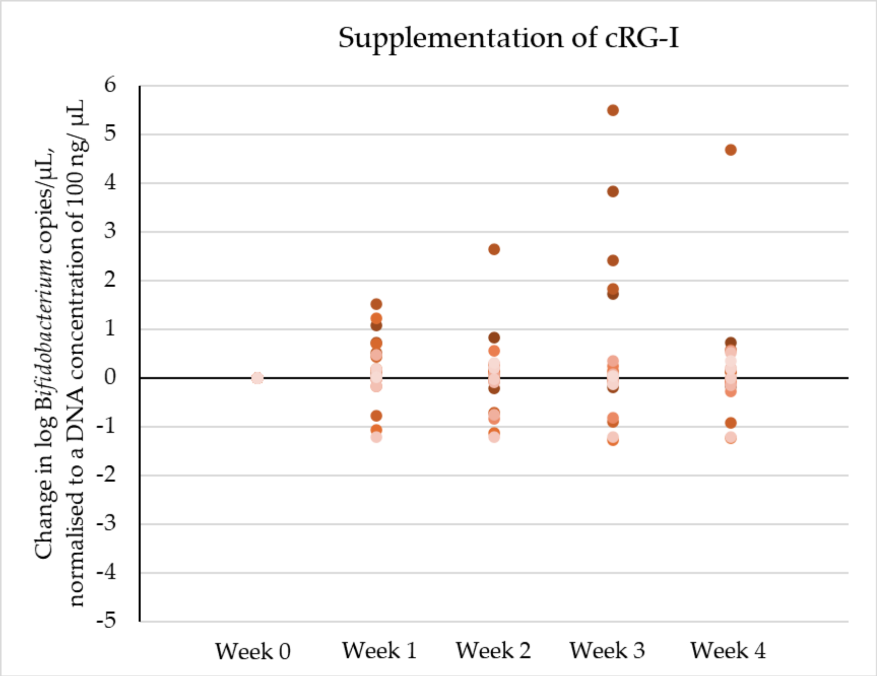

Figure S4. Distribution of individual changes in *Bifidobacterium* counts during four weeks of cRG-I supplementation. Each dot represents one subject, and values are expressed as change in log *Bifidobacterium* copies/µL normalised to a DNA concentration of 100 ng/µL.

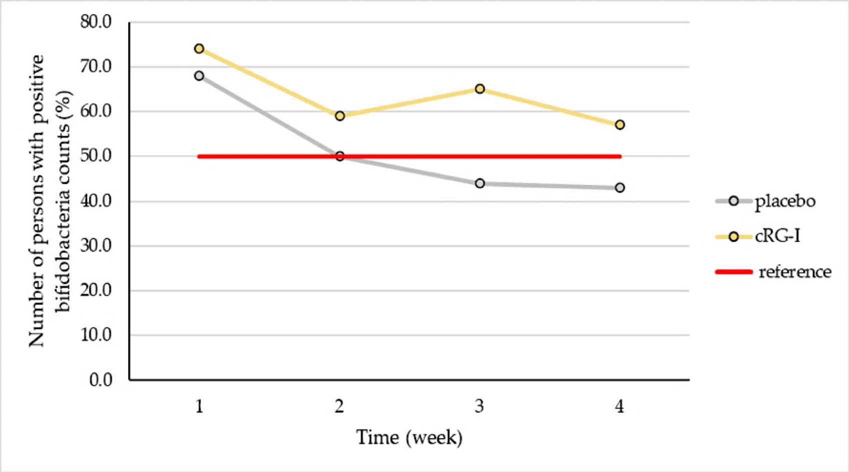

Figure S5. The change in the percentage of persons with an increase in bifidobacteria counts (as compared to the value at the start) during 4 weeks after the start of the consumption of various compounds. Baseline (week 0) was used as the reference point. A person was regarded as “positive” at a given week (week 1–4) if their absolute bifidobacteria counts were higher than at baseline. Each figure point depicts the absolute outcome of at least 24 persons.

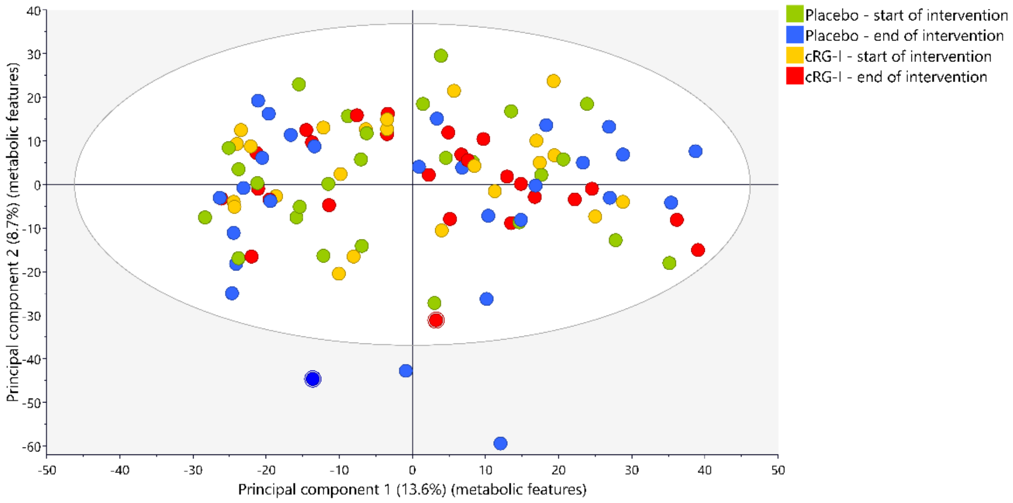

Figure S6. PCA score plot illustrating the potential impact of cRG-I dietary supplementation on LA-REIMS-derived metabolic fingerprints in healthy subjects. No significant metabolic segregation was observed between treatment conditions (Placebo vs cRI-I). Furthermore, supervised modelling using OPLS-DA did not reveal any significant metabolic alterations.

Table S1. Antibodies used for surface staining in flow cytometry (FACS) analysis, including fluorochrome, clone, vendor, catalog number, and volume per test.

**Marker**	**Fluorochrome**	**Clone**	**Vendor**	**Catalog no.**	**µL/Test**	**µL/Mix**
CD66	FITC	TET2	Miltenyi	130-116-522	2	2.0
CD86	PE	FUN-1	BD	557344	1	1.0
HLA-DR	PE/Dazzle	L243	BioLegend	307654	1	1.0
CD123	PerCP-Cy5	7G3	BD	560904	1.25	1.3
BDCA4 (CD304)	PE-Cy7	12C2	BioLegend	354508	5	5.0
CD11c	APC	3.9	BioLegend	301614	1	1.0
CD14	APC-Cy7	M5E2	BioLegend	301820	0.5	0.5
CD16	BV421	3G8	BioLegend	302038	0.5	0.5
CD3	BV510	SP34-2	BD	740187	2	2.0
CD19	BV510	HIB19	BioLegend	302242	1.5	1.5
CD20	BV510	2H7	BioLegend	302340	1.5	1.5
CD56	BV510	B159	BD	740171	1.5	1.5

Staining conditions: 100 µL/tube, 20 min, 4 °C.

Table S2. Effects of cRG-I dietary supplementation on faecal SCFAs and BCFAs (absolute values (μmol/g faeces).

**Faecal Microbial Metabolites**	**Placebo Baseline**	**Placebo Week 4**	***p*-value** Placebo (baseline vs wk 4)	**cRG-I Baseline**	**cRG-I Week 4**	***p*-value** cRG-I (baseline vs wk 4)	***p*-value** placebo vs cRG-I at baseline
Acetic acid (N≥24)	228.97±169.30	258.18±171.28	p=0.387	163.51±99.40	176.63±147.64	p=0.819	p=0.183
Propionic acid (N≥22)	49.20±54.99	57.10±55.92	p=0.137	33.66±36.11	32.90±30.92	p=0.615	p=0.253
Butyric acid (N≥23)	41.79±28.31	46.59±26.35	p=0.278	28.44±18.46	33.38±29.91	p=0.224	p=0.081
Valeric acid (N≥14)	8.08±2.78	9.03±4.72	p=0.187	8.02±3.18	7.82±6.38	p=0.767	p=0.942
Isobutyric acid (N≥14)	7.54±2.68	6.93±2.08	p=0.859	5.96±1.04	6.75±1.91	p=0.034*	p=0.092
Isovaleric acid (N≥16)	8.65±3.51	8.71±3.34	p=0.730	7.56±2.48	8.56±3.82	p=0.122	p=0.446

Data is expressed as mean ± SD. Statistical analysis within each treatment group performed with Wilcoxon signed rank test. Statistical analysis between treatment groups performed with Mann Whitney test. *: statistically significant compared to baseline p<0.05.
